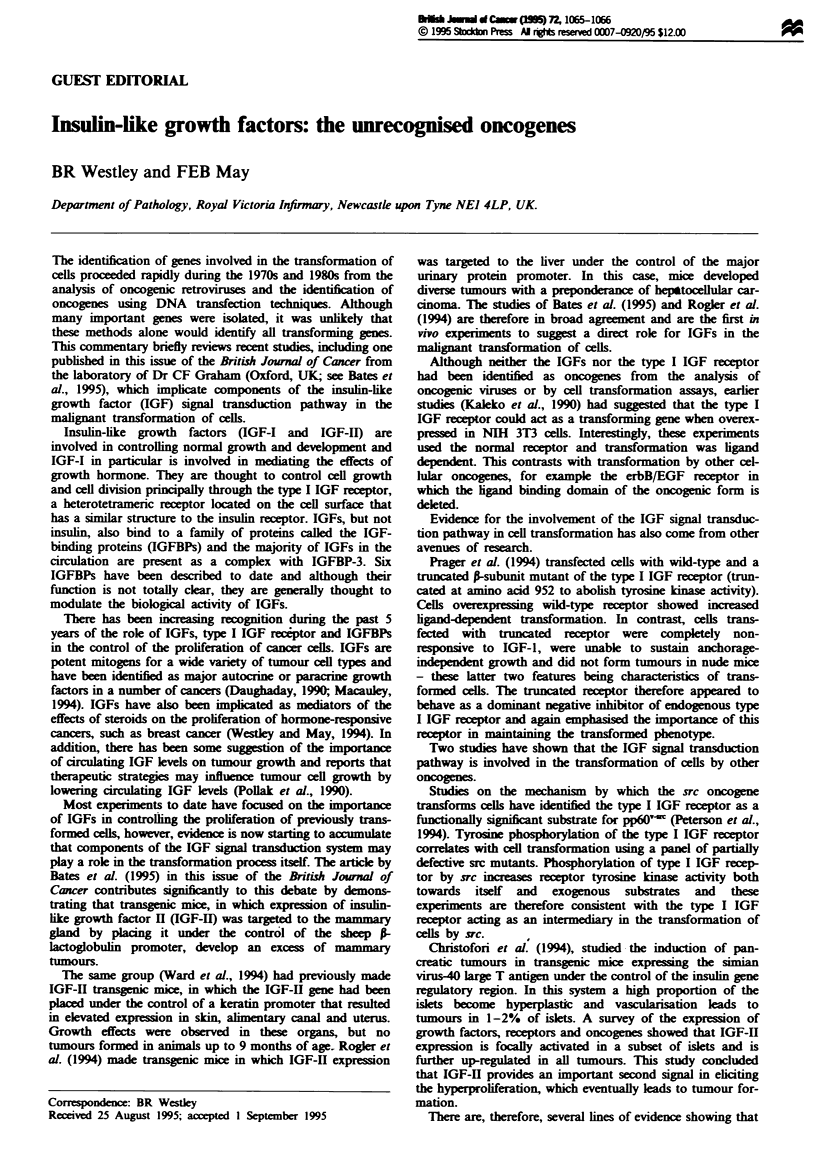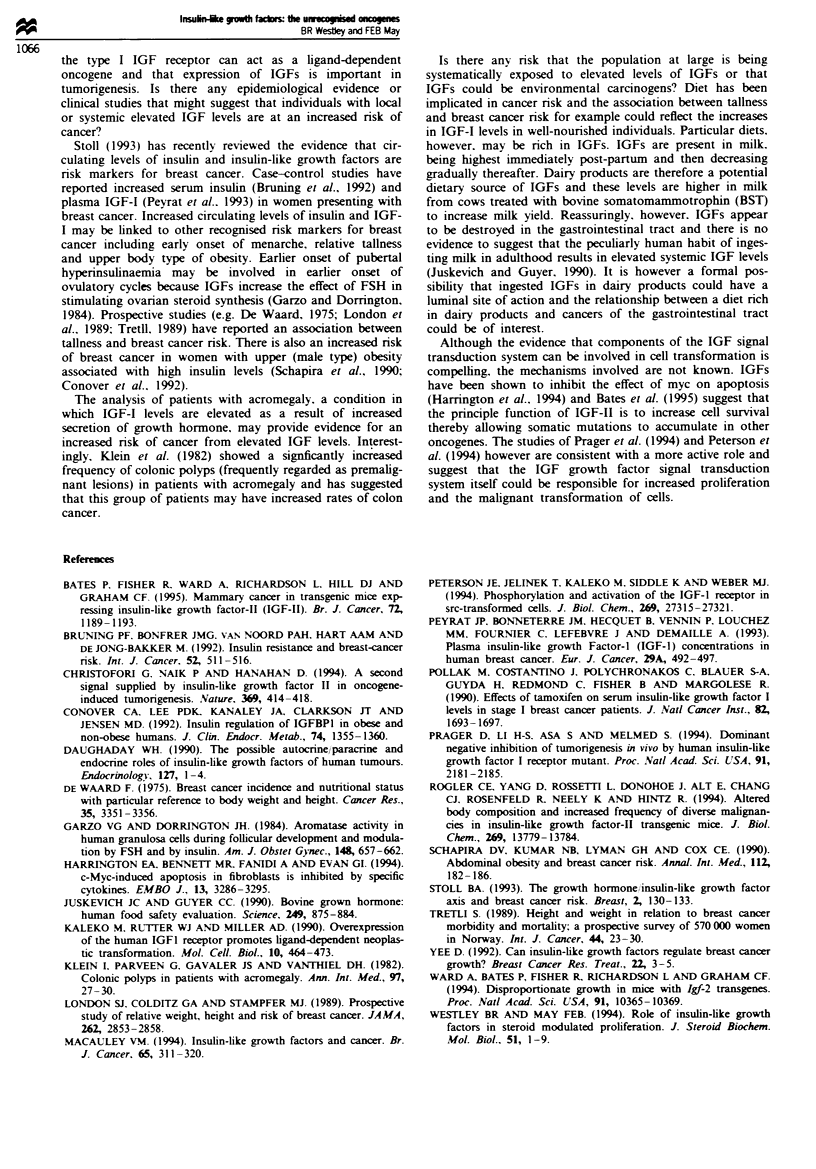# Insulin-like growth factors: the unrecognised oncogenes.

**DOI:** 10.1038/bjc.1995.465

**Published:** 1995-11

**Authors:** B. R. Westley, F. E. May


© 1995 Stockton Press All rights reserved 0007–0920/95 $12.00

GUEST EDITORIAL

### Insulin-like growth factors: the unrecognised oncogenes

BR Westley and FEB May

*Department of Pathology, Royal Victoria Infirmary, Newcastle upon Tyne NE1 4LP, UK.*

The identification of genes involved in the transformation of cells proceeded rapidly during the 1970s and 1980s from the analysis of oncogenic retroviruses and the identification of oncogenes using DNA transfection techniques. Although many important genes were isolated, it was unlikely that these methods alone would identify all transforming genes. This commentary briefly reviews recent studies, including one published in this issue of the *British Journal of Cancer* from the laboratory of Dr CF Graham (Oxford, UK; see Bates *et al.*, 1995), which implicate components of the insulin-like growth factor (IGF) signal transduction pathway in the malignant transformation of cells.

Insulin-like growth factors (IGF-I and IGF-II) are involved in controlling normal growth and development and IGF-I in particular is involved in mediating the effects of growth hormone. They are thought to control cell growth and cell division principally through the type I IGF receptor, a heterotetrameric receptor located on the cell surface that has a similar structure to the insulin receptor. IGFs, but not insulin, also bind to a family of proteins called the IGF-binding proteins (IGFBPs) and the majority of IGFs in the circulation are present as a complex with IGFBP-3. Six IGFBPs have been described to date and although their function is not totally clear, they are generally thought to modulate the biological activity of IGFs.

There has been increasing recognition during the past 5 years of the role of IGFs, type I IGF receptor and IGFBPs in the control of the proliferation of cancer cells. IGFs are potent mitogens for a wide variety of tumour cell types and have been identified as major autocrine or paracrine growth factors in a number of cancers (Daughaday, 1990; Macauley, 1994). IGFs have also been implicated as mediators of the effects of steroids on the proliferation of hormone-responsive cancers, such as breast cancer (Westley and May, 1994). In addition, there has been some suggestion of the importance of circulating IGF levels on tumour growth and reports that therapeutic strategies may influence tumour cell growth by lowering circulating IGF levels (Pollak *et al.*, 1990).

Most experiments to date have focused on the importance of IGFs in controlling the proliferation of previously transformed cells, however, evidence is now starting to accumulate that components of the IGF signal transduction system may play a role in the transformation process itself. The article by Bates *et al.* (1995) in this issue of the *British Journal of Cancer* contributes significantly to this debate by demonstrating that transgenic mice, in which expression of insulin-like growth factor II (IGF-II) was targeted to the mammary gland by placing it under the control of the sheep $\beta$-lactoglobulin promoter, develop an excess of mammary tumours.

The same group (Ward *et al.*, 1994) had previously made IGF-II transgenic mice, in which the IGF-II gene had been placed under the control of a keratin promoter that resulted in elevated expression in skin, alimentary canal and uterus. Growth effects were observed in these organs, but no tumours formed in animals up to 9 months of age. Rogler *et al.* (1994) made transgenic mice in which IGF-II expression was targeted to the liver under the control of the major urinary protein promoter. In this case, mice developed diverse tumours with a preponderance of hepatocellular carcinoma. The studies of Bates *et al.* (1995) and Rogler *et al.* (1994) are therefore in broad agreement and are the first *in vivo* experiments to suggest a direct role for IGFs in the malignant transformation of cells.

Although neither the IGFs nor the type I IGF receptor had been identified as oncogenes from the analysis of oncogenic viruses or by cell transformation assays, earlier studies (Kaleko *et al.*, 1990) had suggested that the type I IGF receptor could act as a transforming gene when overexpressed in NIH 3T3 cells. Interestingly, these experiments used the normal receptor and transformation was ligand dependent. This contrasts with transformation by other cellular oncogenes, for example the erbB/EGF receptor in which the ligand binding domain of the oncogenic form is deleted.

Evidence for the involvement of the IGF signal transduction pathway in cell transformation has also come from other avenues of research.

Prager *et al.* (1994) transfected cells with wild-type and a truncated $\beta$-subunit mutant of the type I IGF receptor (truncated at amino acid 952 to abolish tyrosine kinase activity). Cells overexpressing wild-type receptor showed increased ligand-dependent transformation. In contrast, cells transfected with truncated receptor were completely non-responsive to IGF-1, were unable to sustain anchorage-independent growth and did not form tumours in nude mice – these latter two features being characteristics of transformed cells. The truncated receptor therefore appeared to behave as a dominant negative inhibitor of endogenous type I IGF receptor and again emphasised the importance of this receptor in maintaining the transformed phenotype.

Two studies have shown that the IGF signal transduction pathway is involved in the transformation of cells by other oncogenes.

Studies on the mechanism by which the *src* oncogene transforms cells have identified the type I IGF receptor as a functionally significant substrate for $pp60^{v\text{-}src}$ (Peterson *et al.*, 1994). Tyrosine phosphorylation of the type I IGF receptor correlates with cell transformation using a panel of partially defective src mutants. Phosphorylation of type I IGF receptor by *src* increases receptor tyrosine kinase activity both towards itself and exogenous substrates and these experiments are therefore consistent with the type I IGF receptor acting as an intermediary in the transformation of cells by *src*.

Christofori *et al.* (1994), studied the induction of pancreatic tumours in transgenic mice expressing the simian virus-40 large T antigen under the control of the insulin gene regulatory region. In this system a high proportion of the islets become hyperplastic and vascularisation leads to tumours in 1–2% of islets. A survey of the expression of growth factors, receptors and oncogenes showed that IGF-II expression is focally activated in a subset of islets and is further up-regulated in all tumours. This study concluded that IGF-II provides an important second signal in eliciting the hyperproliferation, which eventually leads to tumour formation.

There are, therefore, several lines of evidence showing that

Correspondence: BR Westley
Received 25 August 1995; accepted 1 September 1995

the type I IGF receptor can act as a ligand-dependent oncogene and that expression of IGFs is important in tumorigenesis. Is there any epidemiological evidence or clinical studies that might suggest that individuals with local or systemic elevated IGF levels are at an increased risk of cancer?

Stoll (1993) has recently reviewed the evidence that circulating levels of insulin and insulin-like growth factors are risk markers for breast cancer. Case-control studies have reported increased serum insulin (Bruning *et al.*, 1992) and plasma IGF-I (Peyrat *et al.*, 1993) in women presenting with breast cancer. Increased circulating levels of insulin and IGF-I may be linked to other recognised risk markers for breast cancer including early onset of menarche, relative tallness and upper body type of obesity. Earlier onset of pubertal hyperinsulinaemia may be involved in earlier onset of ovulatory cycles because IGFs increase the effect of FSH in stimulating ovarian steroid synthesis (Garzo and Dorrington, 1984). Prospective studies (e.g. De Waard, 1975; London *et al.*, 1989; Tretll, 1989) have reported an association between tallness and breast cancer risk. There is also an increased risk of breast cancer in women with upper (male type) obesity associated with high insulin levels (Schapira *et al.*, 1990; Conover *et al.*, 1992).

The analysis of patients with acromegaly, a condition in which IGF-I levels are elevated as a result of increased secretion of growth hormone, may provide evidence for an increased risk of cancer from elevated IGF levels. Interestingly, Klein *et al.* (1982) showed a signficantly increased frequency of colonic polyps (frequently regarded as premalignant lesions) in patients with acromegaly and has suggested that this group of patients may have increased rates of colon cancer.

Is there any risk that the population at large is being systematically exposed to elevated levels of IGFs or that IGFs could be environmental carcinogens? Diet has been implicated in cancer risk and the association between tallness and breast cancer risk for example could reflect the increases in IGF-I levels in well-nourished individuals. Particular diets, however, may be rich in IGFs. IGFs are present in milk, being highest immediately post-partum and then decreasing gradually thereafter. Dairy products are therefore a potential dietary source of IGFs and these levels are higher in milk from cows treated with bovine somatomammotrophin (BST) to increase milk yield. Reassuringly, however, IGFs appear to be destroyed in the gastrointestinal tract and there is no evidence to suggest that the peculiarly human habit of ingesting milk in adulthood results in elevated systemic IGF levels (Juskevich and Guyer, 1990). It is however a formal possibility that ingested IGFs in dairy products could have a luminal site of action and the relationship between a diet rich in dairy products and cancers of the gastrointestinal tract could be of interest.

Although the evidence that components of the IGF signal transduction system can be involved in cell transformation is compelling, the mechanisms involved are not known. IGFs have been shown to inhibit the effect of myc on apoptosis (Harrington *et al.*, 1994) and Bates *et al.* (1995) suggest that the principle function of IGF-II is to increase cell survival thereby allowing somatic mutations to accumulate in other oncogenes. The studies of Prager *et al.* (1994) and Peterson *et al.* (1994) however are consistent with a more active role and suggest that the IGF growth factor signal transduction system itself could be responsible for increased proliferation and the malignant transformation of cells.

### References

BATES P, FISHER R, WARD A, RICHARDSON L, HILL DJ AND GRAHAM CF. (1995). Mammary cancer in transgenic mice expressing insulin-like growth factor-II (IGF-II). *Br. J. Cancer*, **72**, 1189–1193.

BRUNING PF, BONFRER JMG, VAN NOORD PAH, HART AAM AND DE JONG-BAKKER M. (1992). Insulin resistance and breast-cancer risk. *Int. J. Cancer*, **52**, 511–516.

CHRISTOFORI G, NAIK P AND HANAHAN D. (1994). A second signal supplied by insulin-like growth factor II in oncogene-induced tumorigenesis. *Nature*, **369**, 414–418.

CONOVER CA, LEE PDK, KANALEY JA, CLARKSON JT AND JENSEN MD. (1992). Insulin regulation of IGFBP1 in obese and non-obese humans. *J. Clin. Endocr. Metab.*, **74**, 1355–1360.

DAUGHADAY WH. (1990). The possible autocrine/paracrine and endocrine roles of insulin-like growth factors of human tumours. *Endocrinology*, **127**, 1–4.

DE WAARD F. (1975). Breast cancer incidence and nutritional status with particular reference to body weight and height. *Cancer Res.*, **35**, 3351–3356.

GARZO VG AND DORRINGTON JH. (1984). Aromatase activity in human granulosa cells during follicular development and modulation by FSH and by insulin. *Am. J. Obstet Gynec.*, **148**, 657–662.

HARRINGTON EA, BENNETT MR, FANIDI A AND EVAN GI. (1994). c-Myc-induced apoptosis in fibroblasts is inhibited by specific cytokines. *EMBO J.*, **13**, 3286–3295.

JUSKEVICH JC AND GUYER CC. (1990). Bovine grown hormone: human food safety evaluation. *Science*, **249**, 875–884.

KALEKO M, RUTTER WJ AND MILLER AD. (1990). Overexpression of the human IGF1 receptor promotes ligand-dependent neoplastic transformation. *Mol. Cell. Biol.*, **10**, 464–473.

KLEIN I, PARVEEN G, GAVALER JS AND VANTHIEL DH. (1982). Colonic polyps in patients with acromegaly. *Ann. Int. Med.*, **97**, 27–30.

LONDON SJ, COLDITZ GA AND STAMPFER MJ. (1989). Prospective study of relative weight, height and risk of breast cancer. *JAMA*, **262**, 2853–2858.

MACAULEY VM. (1994). Insulin-like growth factors and cancer. *Br. J. Cancer*, **65**, 311–320.

PETERSON JE, JELINEK T, KALEKO M, SIDDLE K AND WEBER MJ. (1994). Phosphorylation and activation of the IGF-1 receptor in src-transformed cells. *J. Biol. Chem.*, **269**, 27315–27321.

PEYRAT JP, BONNETERRE JM, HECQUET B, VENNIN P, LOUCHEZ MM, FOURNIER C, LEFEBVRE J AND DEMAILLE A. (1993). Plasma insulin-like growth Factor-1 (IGF-1) concentrations in human breast cancer. *Eur. J. Cancer*, **29A**, 492–497.

POLLAK M, COSTANTINO J, POLYCHRONAKOS C, BLAUER S-A, GUYDA H, REDMOND C, FISHER B AND MARGOLESE R. (1990). Effects of tamoxifen on serum insulin-like growth factor I levels in stage I breast cancer patients. *J. Natl Cancer Inst.*, **82**, 1693–1697.

PRAGER D, LI H-S, ASA S AND MELMED S. (1994). Dominant negative inhibition of tumorigenesis *in vivo* by human insulin-like growth factor I receptor mutant. *Proc. Natl Acad. Sci. USA*, **91**, 2181–2185.

ROGLER CE, YANG D, ROSSETTI L, DONOHOE J, ALT E, CHANG CJ, ROSENFELD R, NEELY K AND HINTZ R. (1994). Altered body composition and increased frequency of diverse malignancies in insulin-like growth factor-II transgenic mice. *J. Biol. Chem.*, **269**, 13779–13784.

SCHAPIRA DV, KUMAR NB, LYMAN GH AND COX CE. (1990). Abdominal obesity and breast cancer risk. *Annal. Int. Med.*, **112**, 182–186.

STOLL BA. (1993). The growth hormone/insulin-like growth factor axis and breast cancer risk. *Breast*, **2**, 130–133.

TRETLI S. (1989). Height and weight in relation to breast cancer morbidity and mortality; a prospective survey of 570 000 women in Norway. *Int. J. Cancer*, **44**, 23–30.

YEE D. (1992). Can insulin-like growth factors regulate breast cancer growth? *Breast Cancer Res. Treat.*, **22**, 3–5.

WARD A, BATES P, FISHER R, RICHARDSON L AND GRAHAM CF. (1994). Disproportionate growth in mice with *Igf*-2 transgenes. *Proc. Natl Acad. Sci. USA*, **91**, 10365–10369.

WESTLEY BR AND MAY FEB. (1994). Role of insulin-like growth factors in steroid modulated proliferation. *J. Steroid Biochem. Mol. Biol.*, **51**, 1–9.